# Unmanned-Aerial-Vehicle-Assisted Secure Free Space Optical Transmission in Internet of Things: Intelligent Strategy for Optimal Fairness

**DOI:** 10.3390/s24248070

**Published:** 2024-12-18

**Authors:** Fang Xu, Mingda Dong

**Affiliations:** 1 College of Electronic and Information Engineering, Southwest University, Chongqing 400715, China; 2 Qualcomm Communication Technologies (Shanghai) Co., Ltd., Shanghai 201208, China

**Keywords:** secure FSO communication, security authentication, successive interference cancellation (SIC), decoding outage probability, UAV relay

## Abstract

In this article, we consider an UAV (unmanned aerial vehicle)-assisted free space optical (FSO) secure communication network. Since FSO signal is impossible to detect by eavesdroppers without proper beam alignment and security authentication, a BS employs FSO technique to transfer information to multiple authenticated sensors, to improve the transmission security and reliability with the help of an UAV relay with decode and forward (DF) mode. All the sensors need to first send information to the UAV to obtain security authentication, and then the UAV forwards corresponding information to them. Successive interference cancellation (SIC) is used to decode the information received at the UAV and all authenticated sensors. With consideration of fairness, we introduce a statistical metric for evaluating the network performance, i.e., the maximum decoding outage probability for all authenticated sensors. In particular, applying an intelligent approach, we obtain a near-optimal scheme for secure transmit power allocation. With a well-trained allocation scheme, approximate closed-form expressions for optimal transmit power levels can be obtained. Through some numerical examples, we illustrate the various design trade-offs for such a system. Additionally, the validity of our approach was verified by comparing with the result from exhaustive search. In particular, the result with DRL was only 0.3% higher than that with exhaustive search. These results can provide some important guidelines for the fairness-aware design of UAV-assisted secure FSO communication networks.

## 1. Introduction

Secure information transmission has been widely investigated in the past, e.g., [1,2,3,4,5], where complicated techniques needed to be used to avoid information leakage. Since eavesdroppers cannot directly receive an optical signal without beam alignment and security authentication, FSO communication is a promising technique for secure information transmission. Compared to electromagnetic wave transmission, there are several advantages for such technique, e.g., high security performance, high data rate, long transmission distance, and low interference, etc. However, optical signals only can be transferred over a line-of-sight (LoS) path, where the signal quality will considerably deteriorate if obstacles exist in the transmission link. With the rapid development of wireless communications, longer transmission distance and higher reliability are more and more desirable for real applications. While noting that a relay can considerably improve communication coverage and reliability, FSO relaying transmission attracted a lot of attentions from both industry and academia over the past decade.

### 1.1. Previous Work

FSO relaying transmission has been extensively investigated by many researchers in the past, e.g., [6,7,8,9,10,11]. Refs. [6,7,8,9,10,11] mainly focused on mixed FSO/RF transmission. More specifically, Ref. [6] considered a dual-hop multi-user relay system with mixed FSO/RF links, where closed-form expressions for the outage probability and ergodic capacity were derived accordingly. Ref. [7] considered a FSO backhaul network, where concise mathematical expressions for different performance metrics were derived accordingly, e.g., outage probability, average bit error rate (BER), and so on. Ref. [8] considered a mixed FSO/RF spectrum sharing network, where the transmit power and jamming power of a second user (SU) were jointly optimized to achieve the best secrecy performance under both a RF-dominant case and FSO-dominant case. Ref. [9] considered a mixed RF/FSO dual-hop transmission system, where closed-form expressions for outage probability and bit error probability were derived. Ref. [10] analyzed the secrecy outage probability for a dual-hop relaying transmission system involving a hybrid MIMO RF/FSO link, where four transmit antenna selection (TAS) schemes were proposed to enhance the secrecy performance under imperfect CSI. Ref. [11] investigated the throughput maximization issue in a parallel hybrid RF/FSO channel, where the optimal relay selection and time allocation policies were obtained a for buffer-aided relay and non-buffer-aided relay. However, since RF transmission has broadcasting characteristics, all sensors and eavesdroppers can still receive RF signals from an UAV, even with security authentication, and a more complicated technique is needed to ensure the privacy of information transmissions. Combining the above illustrations of the FSO technique and [12], the security performance of mixed FSO/RF transmission is usually not as good as FSO transmission alone.

Refs. [13,14,15,16,17,18,19,20,21,22,23] mainly considered a single FSO relay transmission with high security performance. Ref. [13] considered a three-node cooperative FSO transmission network with an energy harvesting (EH) decode-and-forward (DF) relay, where a novel harvest–store–use (HSU)-based relaying transmission strategy was proposed to improve the overall performance. Ref. [14] considered a single FSO-based distinct eavesdropper (ED) located near the relay, wiretapping the FSO link, where closed-form expressions for the secrecy outage probability (SOP) were derived for three different application scenarios. Ref. [15] investigated secure communications with the aid of hybrid FSO/RF links in a two-phase uplink transmission, where the trajectory and power allocation of the UAV were jointly optimized to maximize the average secrecy rate of the network. Ref. [16] investigated the physical layer security (PLS) performance of a decode and forward (DF) dual-hop mixed FSO/RF communication network in the presence of multiple eavesdroppers, where the lower bound for the secrecy outage probability (SOP) and effective secrecy throughput (EST) were derived. Ref. [17] considered a FSO communication network with a single relay, where a distance-dependent cooperative scheme was proposed to achieve a higher diversity order. Ref. [18] considered a relay-based FSO communication network, where a novel technique was further proposed to regenerate the optical signal. Ref. [19] considered a FSO communication system and proposed a novel protocol for enhancing the cooperative diversity, where a closed-form asymptotic expression for outage probability was derived. Ref. [20] considered a dynamic cooperative FSO transmission network, where two resource-allocation schemes were proposed to jointly optimize the performance. Ref. [21] proposed a novel relay selection strategy to improve the quality of transmission in a multi-relay assisted FSO transmission network, where the closed-form outage probability was derived accordingly. Ref. [22] proposed a novel three-stage methodology for a cooperative FSO transmission network, where closed-form expressions for the conditional error probability were derived. Ref. [23] considered MIMO relay-assisted FSO communication, where the performance of different methods was compared via outage probability analysis in the case of independent fading among the different apertures of communication nodes. Note that all the above references either derived closed-form expressions or used iterative algorithms to optimize the performance. While noting that the optimization problem for fairness-aware secrecy is extremely complex, the approaches adopted in these references are not suitable for optimizing such a  metric.

Since an UAV can be flexibly deployed in some hard-to-reach locations, they have recently been applied to FSO communication networks to forward information [24,25,26,27]. Ref. [24] demonstrated that the usage of a UAV relay can considerably improve the performance of conventional FSO communication networks. In [25,26,27], the performance of UAV-aided FSO relaying transmission networks was analyzed and optimized. However, to the best of our knowledge, an optimal fairness-aware design scheme for a UAV-assisted secure FSO relay communication network has not been fully investigated to date.

### 1.2. Contributions

In this article, we consider an UAV-assisted secure FSO relay communication network, where a BS transmits information to multiple remote sensors with the help of an UAV relay. More specifically, the UAV can effectively decode the received information using SIC, and then forwards it to multiple remote sensors. All the sensors adopt SIC for information decoding. We derive the closed-form expression for decoding outage probability at all sensors. We employ a DRL approach to arrive at a near-optimal scheme, for transmit power allocation, to minimize the maximum decoding outage probability for all sensors. Accordingly, approximate closed-form expressions for optimal transmit power levels can be obtained. The main contributions of our paper are summarized  as below:

- We analyzed the decoding outage probability for all sensors, in terms of an UAV-assisted FSO relay communication network, where all closed-form expressions were derived.
- We used a DRL-based approach to tain the optimal scheme, for transmit power allocation, to minimize the maximum decoding outage probability of all sensors. Note that the relationship between input and output can be modeled using a well-trained policy network, approximate analytical expressions for optimal transmit power levels can be obtained. To the best of our knowledge, this is the first work to investigate an optimal transmission scheme for UAV-assisted FSO communication network in the IoT from the perspective of fairness.
- We illustrate some design trade-offs and compare various design approaches through selected numerical examples. These results can greatly facilitate the fairness-aware design of UAV-assisted FSO communication networks in the IoT.

The rest of this paper is organized as follows. Section 2 presents the system and FSO channel model under consideration. The closed-form outage probabilities for all authenticated sensors are derived in Section 3. Section 4 addresses the issue of parameter optimization in terms of fairness-aware outage probability minimization. After that, we present some selected numerical examples and discuss some design trade-offs in Section 5. Finally, some concluding remarks are presented in Section 6.

## 2. System and Channel Model

### 2.1. System Model

**Figure 1:**
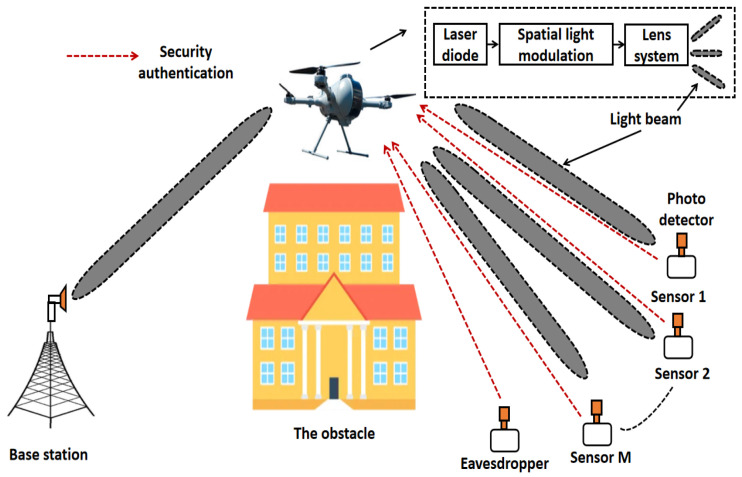
Configuration of UAV-assisted FSO communication network.

We consider a secure FSO relaying communication network, comprising of one BS, one UAV relay, one eavesdropper, and multiple sensors, as shown in Figure 1. Since the complexity of implementing a wavelength division multiplex (WDM) is extremely high, we use authentication-based non-orthogonal multiple access (NOMA) to simultaneously transmit information to multiple sensors in this case. More specifically, the BS first transfers data to the UAV. After that, the UAV decodes the received information and forwards it to all sensors. An optical phased array can be used to facilitate the alignment and tracking of an optical beam [28], to perform accurate dynamic alignment for FSO communication. Note that all the sensors need to send signal to the UAV for security authentication, and then they are authorized to access the network for information transmission and reception. An eavesdropper also requires security authentication from the UAV, as a normal sensor. We assume that the probability that the UAV identifies an eavesdropper as a normal sensor is $P_{secr}$, which is also regarded as the secrecy outage of data transmission. SIC is used to decode the received information at the UAV and all sensors. According to [29], an UAV can employ spatial light modulation and a lens system to generate multiple light beams to point at authenticated sensors. Such a system is very simple to realize in real application scenarios. In addition, we ignore the transfer efficiency from the electrical signal to optical signal, and direct optical link is supposed to be not available due to the existence of obstacles. Within the first phase of the information transmission, the received signal of the UAV is denoted by

(1) $$\begin{array}{c} y_{r}\left(t\right)=\left(s_{1}\left(t\right)+s_{2}\left(t\right)+\cdots +s_{M}\left(t\right)\right)\sqrt{I_{SR}}+N_{R}\left(t\right), \end{array}$$

where $I_{SR}$ denotes the channel power gain of the FSO link from the BS to the UAV, $s_{i}\left(t\right)$ denotes the transmitted signal for sensor *i*, i=1,2,⋯,M, and $N_{R}\left(t\right)$ denotes the received noise signal at the UAV. Within the second phase of information transmission, the received signal of sensor *i*, i=1,2,⋯,M, is given by

(2) $$\begin{array}{c} y_{D_{i}}\left(t\right)=s_{i}\left(t\right)\sqrt{I_{RD_{i}}}+N_{D_{i}}\left(t\right), \end{array}$$

where $I_{RD_{i}}$ denotes the channel power gain of the FSO link from the UAV to sensor *i*, and $N_{D_{i}}\left(t\right)$ denotes the noise signal received at sensor *i*.

### 2.2. Channel Model

We consider a composite optical channel model [30], where the channel power gain is proportional to the path loss, atmospheric turbulence, and pointing error. Specifically, the overall channel power gain $I_{SR}$ can be calculated by

(3) $$\begin{array}{c} I_{SR}=I_{SR}^{l}I_{SR}^{p}I_{SR}^{a}. \end{array}$$

Here, $I_{SR}^{l}$ denotes the power gain of the path loss, $I_{SR}^{P}$ denotes the power gain of the pointing error, and $I_{SR}^{a}$ denotes the power gain of atmosphere turbulence following a gamma–gamma distribution. In particular, the PDF (probability density function) of $I_{SR}$ is shown as

(4) $$\begin{array}{cc} \; & f_{I_{SR}}\left(x\right)=\frac{\alpha_{SR}\beta_{SR}\epsilon_{SR}^{2}}{I_{SR}^{l}A_{SR}\Gamma \left(\alpha_{SR}\right)\Gamma \left(\beta_{SR}\right)}G_{1,3}^{3,0}\left[\frac{\alpha_{SR}\beta_{SR}}{I_{SR}^{l}A_{SR}}x{|}_{\epsilon_{SR}^{2}-1,\alpha_{SR}-1,\beta_{SR}-1}^{\epsilon_{SR}^{2}}\right]. \end{array}$$

Here, $G_{1,3}^{3,0}\left[.\right]$ denotes the Meiger-G-function, $\alpha_{SR}$ and $\beta_{SR}$ are parameters related to the atmosphere turbulence, while $A_{SR}$ and $\epsilon_{SR}$ are parameters related to the pointing error. $I_{SR}^{l}$ is denoted by

(5) $$\begin{array}{c} I_{SR}^{l}=\exp\left(-kL_{SR}\right), \end{array}$$

where *k* denotes the attenuation coefficient, and $L_{SR}$ denotes the distance from the BS to the UAV.

In terms of the second-hop links, following a similar analysis process, the PDF of $I_{RD_{i}}$, i=1,2,⋯,M can be denoted by

(6) $$\begin{array}{c} f_{I_{RD_{i}}}\left(x\right)=Q_{RD_{i}}G_{1,3}^{3,0}\left[\frac{\alpha_{RD_{i}}\beta_{RD_{i}}}{I_{RD_{i}}^{l}A_{RD_{i}}}x{|}_{\epsilon_{RD_{i}}^{2}-1,\alpha_{RD_{i}}-1,\beta_{RD_{i}}-1}^{\epsilon_{RD_{i}}^{2}}\right], \end{array}$$

where $Q_{RD_{i}}$ denotes $\frac{\alpha_{RD_{i}}\beta_{RD_{i}}\epsilon_{RD_{i}}^{2}}{I_{RD_{i}}^{l}A_{RD_{i}}\Gamma \left(\alpha_{RD_{i}}\right)\Gamma \left(\beta_{RD_{i}}\right)}$. Here, $\alpha_{RD_{i}}$, $\beta_{RD_{i}}$, $\epsilon_{RD_{i}}$, $I_{RD_{i}}$ and $I_{RD_{i}}^{l}$ are the channel parameters for link from the UAV to sensor *i*, i=1,2,⋯,M, which have the same physical meaning as $\alpha_{SR}$, $\beta_{SR}$, $\epsilon_{SR}$, $I_{SR}$ and $I_{SR}^{l}$ in the first-hop link. Then, the CDF of $I_{RD_{i}}$ can be derived as follows:

(7) $$\begin{array}{cc} \; & F_{I_{RD_{i}}}\left(x\right)=Q_{RD_{i}}xG_{2,4}^{3,1}\left[\frac{\alpha_{RD_{i}}\beta_{RD_{i}}}{I_{RD_{i}}^{l}A_{RD_{i}}}x{|}_{\epsilon_{RD_{i}}^{2}-1,\;\alpha_{RD_{i}}-1,\;\beta_{RD_{i}}-1,\;-1}^{0,\;\epsilon_{RD_{i}}^{2}}\right]. \end{array}$$

## 3. Outage Probability Analysis

In this section, we analyze the decoding outage probability at each sensor, where the transmit power level of $s_{i}\left(t\right)$ is denoted by $P_{i}$, i=1,2,⋯,M. We assume that $P_{1}\;\geq \;P_{2}\;\geq \;\cdots \;\geq \;P_{M}$. Note that different from conventional SIC, the order of the transmit power levels is not only determined based on the order of magnitude of the channel power gains, where the optimal scheme for transmit power allocation is obtained using the DRL approach. Combined with the descriptions in Section 2, while noting that an imperfect SIC scheme is adopted for decoding the signals in a descending order of transmit power levels, the received signal-to-interference-and-noise ratio (SINR) of the UAV for decoding $s_{i}\left(t\right)$ is calculated by

(8) $$\begin{array}{cc} \; & SINR_{R,i}=\frac{P_{i}}{\left(P_{i+1}+\cdots +P_{M}\right)+u\left[i-2\right]\xi \left(P_{1}+\cdots +P_{i-1}\right)+\sigma^{2}/I_{SR}}. \end{array}$$

Here, ξ denotes the proportional coefficient of residual interference caused by imperfect decoding [31], u[.] denotes the unit step function. Using $\gamma_{th}$ to denote the threshold of the SINR for successful information decoding, the outage probability of decoding $s_{i}\left(t\right)$ can be denoted by

(9) $$\begin{array}{cc} \; & 1-\prod_{k=1}^{i}\left(1-\mathrm{Pr}\left[SINR_{R,k}<\gamma_{th}\right]\right)=\\ \; & 1-\prod_{k=1}^{i}\left(1-\frac{Q_{k}\epsilon_{SR}^{2}G_{2,4}^{3,1}\left[Q_{k}{|}_{\epsilon_{SR}^{2}-1,\;\alpha_{SR}-1,\;\beta_{SR}-1,\;-1}^{0,\;\epsilon_{SR}^{2}}\right]}{\Gamma \left(\alpha_{SR}\right)\Gamma \left(\beta_{SR}\right)}\right), \end{array}$$

where $Q_{k}$ denotes $\frac{\alpha_{SR}\beta_{SR}\gamma_{th}\sigma^{2}/\left(A_{SR}I_{SR}^{l}\right)}{P_{k}-\gamma_{th}\left(P_{k+1}+\cdots +P_{M}+u\left[k-2\right]\xi \left(P_{1}+\cdots +P_{k-1}\right)\right)}$. $\mathrm{Pr}[SINR_{R,k}<\gamma_{th}]$ can be calculated by performing an integral operation for $f_{I_{SR}}\left(x\right)$ from 0 to $A_{SR}I_{SR}^{l}Q_{k}/\left(\alpha_{SR}\beta_{SR}\right)$. The corresponding integral result can be obtained based on Equation (26) of [32]. During the second-hop transmission from the UAV to all sensors, the UAV utilizes spatial modulation to generate multiple directional light beams to point at the corresponding sensors [29]. Applying the imperfect SIC, in the  case that the eavesdropper does not obtain security authentication from the UAV, the received SINR of sensor *j*, j=1,2,⋯,M, for decoding $s_{i}\left(t\right)$ can be denoted by

(10) $$\begin{array}{cc} \; & SINR_{D_{j\to i}}=\frac{P_{i}}{P_{i+1}+\cdots +P_{M}+u\left[i-2\right]\xi \left(P_{1}+\cdots +P_{i-1}\right)+\frac{\sigma^{2}}{I_{RD_{j}}}}. \end{array}$$

Accordingly, the resulting outage probability at sensor *j* for decoding $s_{i}\left(t\right)$ is derived as below

(11) $$\begin{array}{c} \mathrm{Pr}\left[SINR_{D_{j\to i}}<\gamma_{th}\right]=\int_{0}^{Q_{RD}^{i}}f_{I_{RD_{j}}}\left(x\right)dx=F_{I_{RD_{j}}}\left(Q_{RD}^{i}\right), \end{array}$$

where $Q_{RD}^{i}$ denotes $\frac{\gamma_{th}\sigma^{2}}{P_{i}-\gamma_{th}\left(P_{i+1}+\cdots +P_{M}+u\left[i-2\right]\xi \left(P_{1}+\cdots +P_{i-1}\right)\right)}$. Note that a decoding outage will occur in cases where either the received SINR of the first-hop link or that of the second-hop link is not high enough for the decoding the information properly. As such, the decoding outage probability for sensor *i*, i=1,2,⋯,M, can be calculated as follows

(12) $$\begin{array}{cc} \; & P_{out,i}=1-\left(1-P_{secr}\right)\prod_{q=1}^{i}\mathrm{Pr}\left[SINR_{D_{i\to q}}\geq \gamma_{th}\right]\prod_{k=1}^{i}\mathrm{Pr}\left[SINR_{R,k}\geq \gamma_{th}\right]. \end{array}$$

Combining Equations (9) and (11), a closed-form expression for Equation (12) can be derived accordingly. Note that a secrecy outage with the probability of $P_{secr}$ is also regarded as a decoding outage in this case, where the eavesdropper also obtains the security authentication from the UAV.

## 4. Fairness-Aware Optimal Transmission Scheme

In this section, we will investigate the optimal transmission scheme of an UAV-assisted FSO communication network from the perspective of fairness. We apply a DRL-based approach to arrive at a near-optimal allocation scheme for the transmit power levels. In particular, with a well-trained policy, while noting that a deep neural network (DNN) can model the relationship between the input and output, approximate closed-form expressions for near-optimal transmit power levels can be obtained.

To ensure the fairness among all authenticated sensors, an  objective function is set to their maximum decoding outage probability. Note that fairness performance becomes better when maximum decoding outage probability decreases. Combining all the above analyses, the optimization problem is formulated as follows

$$\begin{array}{cc} \; & \min_{P_{1},\;P_{2},\;\cdots ,\;P_{M}}\;\max\left\{P_{out,1},\;P_{out,2},\;\cdots ,\;P_{out,M}\right\},\\ \; & \begin{array}{ccc} s.t.\; & 0<P_{1}+P_{2}+\cdots +P_{M}\leq P_{\max},\;0 & <P_{1},\;\cdots ,\;0<P_{M}. \end{array} \end{array}$$

Here, $P_{out,i}$ was derived in Section 3, i=1,2,⋯,M. We can see that such an objective function is extremely complex and not convex with respect to all variables. Obviously, it is impossible to derive closed-form optimal solutions in this case. Note that the FSO channel parameters are unstable under dynamic weather conditions. With iterative algorithms, the optimization process needs to be repeated when the model parameters change, which will lead to a very high computational complexity. In addition, the objective function involves a complicated Meiger-G function, which does not have a gradient expression. As such, the gradient ascent approach is not feasible for handling the corresponding optimization issues. As such, we intend to use the DRL approach to arrive at a near-optimal policy model for approximating the closed-form expressions of the optimal transmit power levels. The state space S is defined as $\left\{\alpha_{SR},\;\beta_{SR},\;\alpha_{RD_{1}},\;\beta_{RD_{1}},\;\alpha_{RD_{2}},\;\beta_{RD_{2}},\cdots ,\;\alpha_{RD_{M}},\;\beta_{RD_{M}}\right\}$, and the action space R is defined as $\left\{P_{1},\;P_{2},\;\cdots ,\;P_{M}\right\}$.

### 4.1. Reward Function

Since our objective is to minimize the maximum decoding outage probability for all sensors, we define the inverse of the above objective function as the reward function, shown as follows

(13) $$\begin{array}{c} R_{statis}=\frac{1}{\max\{P_{out,1},\;P_{out,2},\;\cdots ,\;P_{out,M}\}}. \end{array}$$

### 4.2. Deep Actor Networks and Critic Network

**Figure 2:**
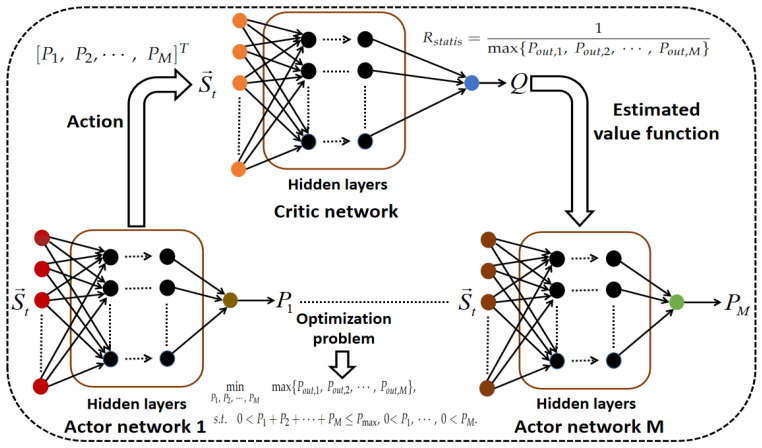
The configuration of the employed deep neural networks.

In this case, through interacting with one critic network, several actor networks can be well-trained to determine the optimal actions under a dynamic state input. Since the gradient operation of the reward function may be NAN for some random experience tuples, we introduce a critic network to approximate it during the training process. As shown in Figure 2, the critic network is used to output approximate reward value for a specific power allocation scheme, and the actor networks are used to determine the transmit power levels of all sensors. Here, the ReLu function is used as the output activation function. The parameter set of the critic network is denoted by $\theta^{Q}$, and the parameter set of the actor network *k* is denoted by $\theta^{\mu_{k}}$, k=1,2,⋯,M. The input of all actor networks is the statistical channel state information ${\vec{s}}_{t}$, which is defined as ${\left[\alpha_{SR},\;\beta_{SR},\;\alpha_{RD_{1}},\;\beta_{RD_{1}},\;\alpha_{RD_{2}},\;\beta_{RD_{2}},\cdots ,\;\alpha_{RD_{M}},\;\beta_{RD_{M}}\right]}^{T}$. The input of the critic network consists of a state vector ${\vec{s}}_{t}$ and action vector ${\vec{a}}_{t}$, where ${\vec{a}}_{t}$ is denoted by ${\left[P_{1},\;P_{2},\cdots ,\;P_{M}\right]}^{T}$. More specifically, within each training iteration, the critic network can evaluate the output of the actor networks and then feed back an estimated reward value to them, based on which all the actor networks can improve their performance by performing gradient ascent. Also note that we use $Q(\vec{s_{t}},\;\vec{a_{t}}|\theta^{Q})$ to denote the output of the critic network and use $\mu_{k}\left(\vec{s_{t}}|\theta^{\mu_{1}}\right)$ to denote the output of the actor network *k*, k=1,2,⋯,M, respectively. After a certain amount of iterations, all actor networks can be properly trained. While noting that the exact mathematical relationship between the input and output can be modeled by a deep neural network, approximate closed-form expressions for optimal transmit power levels can be obtained from well-trained actor networks.

### 4.3. Model Training Process

Prior to the beginning of training, we need to set up a memory buffer with the size of *K* to save the experience tuples. In particular, we randomly generate a statistical channel state vector ${\vec{s}}_{t}={\left[\alpha_{SR},\;\beta_{SR},\;\alpha_{RD_{1}},\;\beta_{RD_{1}},\cdots ,\;\alpha_{RD_{M}},\;\beta_{RD_{M}}\right]}^{T}$, and then feed it to the actor networks to arrive at an action vector ${\vec{a}}_{t}={\left[P_{1},\cdots ,\;P_{M}\right]}^{T}$. By substituting the obtained state vector and action vector into Equation  (13), the resulting reward value $R_{t}$ can be calculated accordingly. The obtained new experience tuple $\vec{a_{t}}$-$\vec{s_{t}}$-$R_{t}$ is put into the memory buffer. Note that such a process needs to be repeated several times, until the memory buffer is full.

Once *K* experience tuples are available, we can start the training process. During each iteration, following a similar process described above, we first generate a new state–action–reward tuple and put it into the memory buffer to randomly replace an existing tuple. After that, we extract *N* experience tuples at random from the memory buffer and use them to construct a critic network for approximating the reward function. The parameter set of the critic network can be updated by minimizing the loss function, shown as follows

(14) $$\begin{array}{c} \theta^{Q^{\prime }}=\min_{\theta^{Q}}\sum_{i=1}^{N}{\left(Q\left(\vec{s_{i}},\;\vec{a_{i}}|\theta^{Q}\right)-R_{i}\right)}^{2}. \end{array}$$

After obtaining an updated critic network, we can further renew the actor networks by performing the operation of joint gradient ascent with the help of chain rule. The parameter set of actor network *j*, j=1,2,⋯,M, is updated as follows

(15) $$\begin{array}{cc} \; & \theta^{\mu_{j}^{\prime }}\leftarrow \theta^{\mu_{j}}+\frac{\epsilon }{N}\sum_{i=1}^{N}{▽}_{\mu_{j}\left(\vec{s_{i}}|\theta^{\mu_{j}}\right)}Q\left(\vec{s},\vec{a}|\theta^{Q^{\prime }}\right){|}_{\vec{s}=\vec{s_{i}},\vec{a}=\vec{a_{i}}}{▽}_{\theta^{\mu_{j}}}\mu_{j}\left(\vec{s_{i}}|\theta^{\mu_{j}}\right), \end{array}$$

where

(16) $$\begin{array}{c} \vec{a_{i}}={\left[\mu_{1}\left(\vec{s_{i}}|\theta^{\mu_{1}}\right),\cdots ,\;\mu_{M}\left(\vec{s_{i}}|\theta^{\mu_{M}}\right)\right]}^{T}. \end{array}$$

Here, ϵ denotes the learning rate for all actor networks.

### 4.4. Random Exploration

To increase the chance of converging to a global optimal action policy, we introduce random exploration into all output actions. More specifically, the output of the actor network *k*, k=1,2,⋯,M, is denoted by

(17) $$\begin{array}{c} a_{k}=\max\left\{0,\;N\left(\mu \left(\vec{s}|\theta^{\mu_{k}}\right),\;v_{k}\right)\right\}, \end{array}$$

where N(.) denotes the mathematical sign of the normal distribution and $v_{k}$ denotes the corresponding variance, k=1,2,⋯,M. The transmit power level $P_{k}$, k=1,2,⋯,M, can be calculated as below

(18) $$\begin{array}{c} P_{k}=P_{\max}a_{k}/\left(\Sigma_{j=1}^{M}a_{j}\right). \end{array}$$

At the end of each training iteration, the variance $v_{k}$ is updated to $\beta v_{k}$, k=1,2,⋯,M, where β is a constant ranging from 0 to 1. As such, all the variances should become smaller and smaller with on-going training, based on which the scope of exploration is gradually reduced as well. After a sufficient number of training iterations, all actor networks can be well trained to determine the near-optimal scheme for transmit power allocation.

### 4.5. The Pseudo Code

The pseudo code of the action policy training is shown in Algorithm 1.
**Algorithm 1** The pseudo code of training.**for** t ∈[1,2,⋯,T] **do**   Generate a random state vector $\vec{s_{t}}$.   Generate action vector $\vec{a_{t}}$ with given state vector $\vec{s_{t}}$ as in Equations (17) and (18) and the above descriptions.   Use $\vec{a_{t}}$ and $\vec{s_{t}}$ to calculate the resulting reward $R_{t}$ as in Equation (13).   Employ the state–action–reward tuple $\vec{s_{t}}$-$\vec{a_{t}}$-$R_{t}$ to randomly replace an existing tuple in the memory buffer.   Randomly extract *N* state–action–reward tuples $\vec{s_{i}}$-$\vec{a_{i}}$-$R_{i}$, i=1,2,⋯,N, from the memory buffer to update the parameter set of the critic network to $\theta^{Q^{\prime }}$ as in Equation (14).   Update the parameter set of all actor networks by performing joint gradient ascent as in Equation (15).   Update the variance $v_{k}$ to $\beta v_{k}$, k=1,⋯,M.**end for**

## 5. Numerical Results

In this section, we present some selected numerical results to illustrate the effect of the proposed approach. The common parameters, used in the simulation, are shown in Table 1. There are three hidden layers for each actor network, each of which has 16 neurons. As for the critic network, there is only one hidden layer with 128 neurons. To simplify the process, we assume that there are three sensors in the simulation.

**Figure 3:**
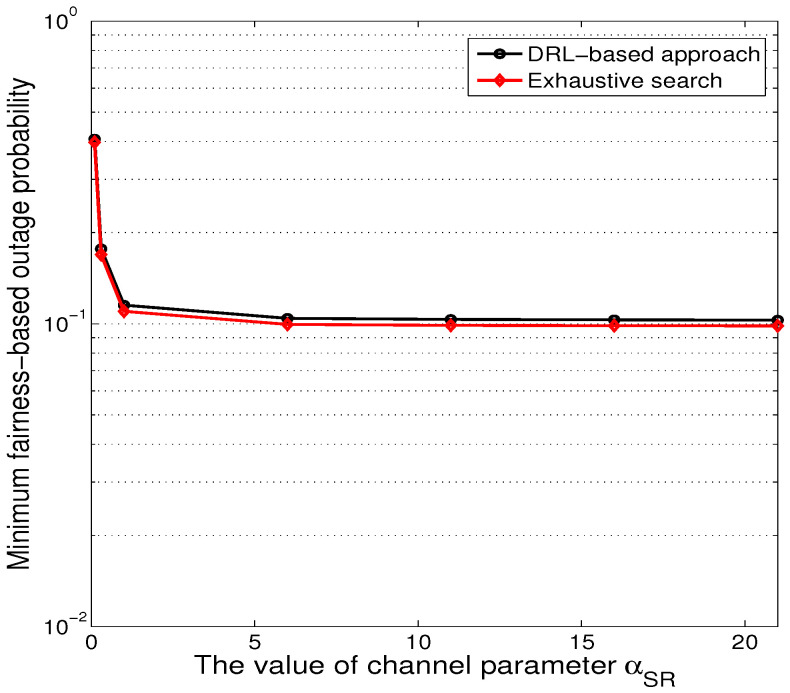
The minimum value for the maximum outage probability for the exhaustive search and deep reinforcement learning, $\beta_{SR}=13$, $\epsilon_{SR}=0.4$, $\alpha_{RD1}$=13, $\beta_{RD1}=7.5$, $\epsilon_{RD1}=1.7$, $\alpha_{RD2}=13.5$, $\beta_{RD2}=8$, $\epsilon_{RD2}=2.1$, $\alpha_{RD3}$=11.5, $\beta_{RD3}=7.2$, $\epsilon_{RD3}=2.4$.

Figure 3 presents the minimum value for the maximum decoding outage probability of all sensors as a function of the first-hop channel parameter $\alpha_{SR}$, where $\alpha_{SR}$ varied from 0.3 to 24. As expected, the minimum value for the maximum decoding outage probability of all sensors from the exhaustive search is very close to that from the DRL approach. The complexity of the proposed DRL approach is mainly from the offline training, which needs around 90,000 iterations. As for the online operation, the well-trained deep network only needs to output the action, without involving any iterations, where the complexity is very low. As such, we can show that our proposed approach has arrived at near-optimal performance.

**Figure 4:**
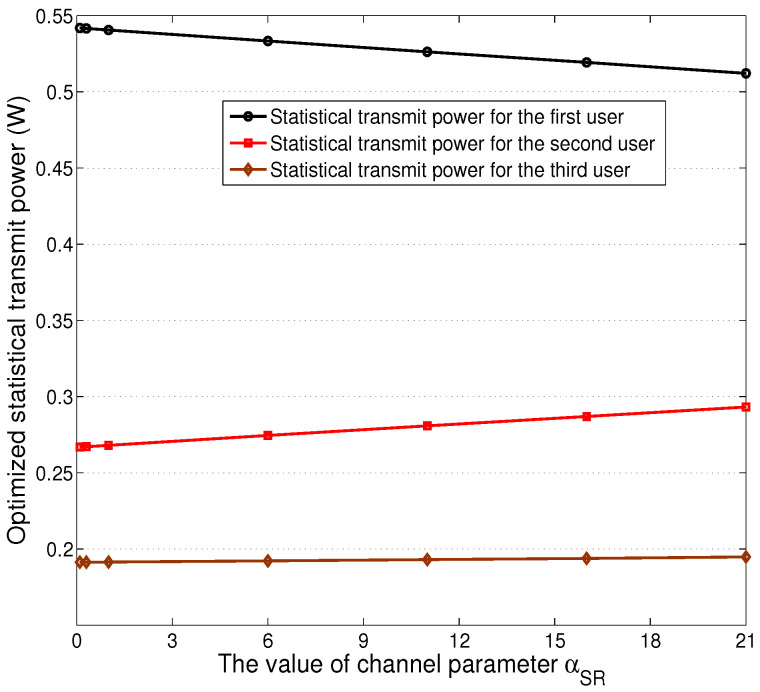
Near-optimal statistical transmit power levels for the signals of all machines, $\beta_{SR}=13$, $\epsilon_{SR}=0.4$, $\alpha_{RD1}$=13, $\beta_{RD1}=7.5$, $\epsilon_{RD1}=1.7$, $\alpha_{RD2}=13.5$, $\beta_{RD2}=8$, $\epsilon_{RD2}=2.1$, $\alpha_{RD3}$=11.5, $\beta_{RD3}=7.2$, $\epsilon_{RD3}=2.4$.

Figure 4 presents the statistical transmit power levels of the well-trained actor networks as a function of the first-hop channel parameter $\alpha_{SR}$. We can see that most power is allocated to transfer signal to the first sensor, and the transmit power of the third sensor’s signal has the minimum level. In addition, we can observe that the transmit power level of the first sensor’s signal decreased as $\alpha_{SR}$ increased, while the transmit power levels for other two sensors’ signals have a contrary trend. Note that the sum of all transmit power levels is always equal to $P_{\max}$ with varying $\alpha_{SR}$.

**Figure 5:**
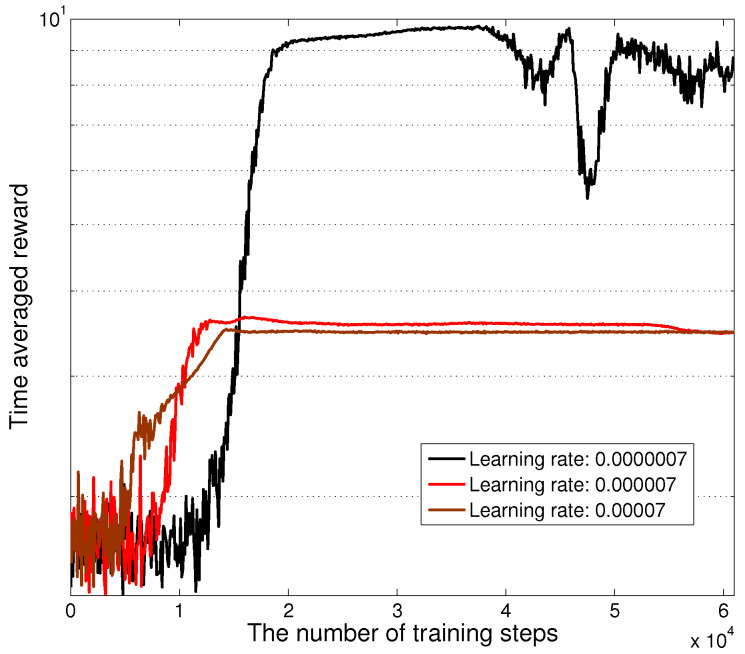
The average reward over 100 consecutive steps for the training of the statistical optimal scheme.

Figure 5 presents the average reward over 100 consecutive steps during the training process of the optimal scheme for different learning rate settings. As expected, we can see that the training reward firstly increases with on-going training and then converges to a stable value. We can also observe that the learning rate of 0.0000007 leads to a considerably higher convergent reward value than those of 0.000007 and 0.00007. As such, it is of great importance to set an appropriate learning rate to achieve good training performance. Note that the fluctuation of the reward function is caused by random exploration, which will approach zero after a certain amount of training iterations.

## 6. Conclusions

In this paper, we considered a UAV-assisted secure FSO communication network, where the BS transfers information to multiple authenticated sensors via an optical link. We analyzed and minimized the maximum decoding outage probability for all authenticated sensors. More specifically, we applied a DRL-based approach to arrive at a near-optimal scheme for transmit power allocation from the perspective of fairness. The validity of our approach was verified by comparing with the result from exhaustive search. These results will be very valuable for improving the fairness of UAV-assisted secure FSO communication networks.

## Figures and Tables

**Table 1:** Parameters used in the simulation.

Notations	Meaning	Values
$\sigma^{2}$	Average noise power	$10^{-5}$ Watt
B	Channel bandwidth	200 KHz
$P_{\max}$	The peak power	1 Watt
*v*	Exploration variance	14
β	Updation factor	0.99
$\gamma_{\mathrm{th}}$	SINR threshold	0.2
$P_{secr}$	security outage probability	0.05

## Data Availability

The raw data supporting the conclusions of this article will be made available by the authors on request.
